# Primary Lung Adenocarcinoma With ALK Gene Rearrangement Mostly Occupied by the Signet-Ring Cell Carcinoma Component: A Case Report

**DOI:** 10.7759/cureus.45068

**Published:** 2023-09-11

**Authors:** Akira Okimura, Hiroshi Hirano, Yumika Ito, Naohiro Kajiwara, Munehide Nakatsugawa

**Affiliations:** 1 Diagnostic Pathology, Tokyo Medical University Hachioji Medical Center, Tokyo, JPN; 2 Thoracic Surgery, Tokyo Medical University Hachioji Medical Center, Tokyo, JPN

**Keywords:** histopathology and immunohistochemistry, alk gene fusion, signet-ring cell carcinoma, primary pulmonary adenocarcinoma, rare case report

## Abstract

Primary lung carcinoma tumors possessing a signet-ring cell carcinoma (SRCC) component at varying proportions are rare, while those primarily composed of an SRCC component are much rarer. Reported here is a case of primary lung adenocarcinoma primarily composed of an SRCC component with a scant acinar component that developed in an 81-year-old male. Approximately 95% of the adenocarcinoma was occupied by an SRCC component that was shown to be diastase-resistant based on positive periodic acid-Schiff staining. Immunostaining for *ALK* and fluorescence in situ hybridization analysis (break-apart assay) showed the presence of an *ALK* gene rearrangement. Findings in this case indicated a primary lung adenocarcinoma with *ALK *gene rearrangement, in which an SRCC component accounted for approximately 95% of the tumor.

## Introduction

Previous studies have noted a few primary lung carcinomas that contained signet-ring cell carcinoma (SRCC) components at varying proportions [1-5], with rates of incidence ranging from 0.14% to 2.0% of all examined cases. In those reports, the SRCC component proportion ranged from 5% to 100%, though that was greater than 90% in only five of the tumors, indicating that a primary lung carcinoma occupied primarily by an SRCC component is quite rare. Such occurrences have been reported to be strongly associated with *ALK* gene rearrangements [6]. Here, we report a case of primary lung adenocarcinoma with *ALK* gene rearrangement, in which an SRCC component occupied approximately 95% of the tumor.

## Case presentation

**Figure 1:**
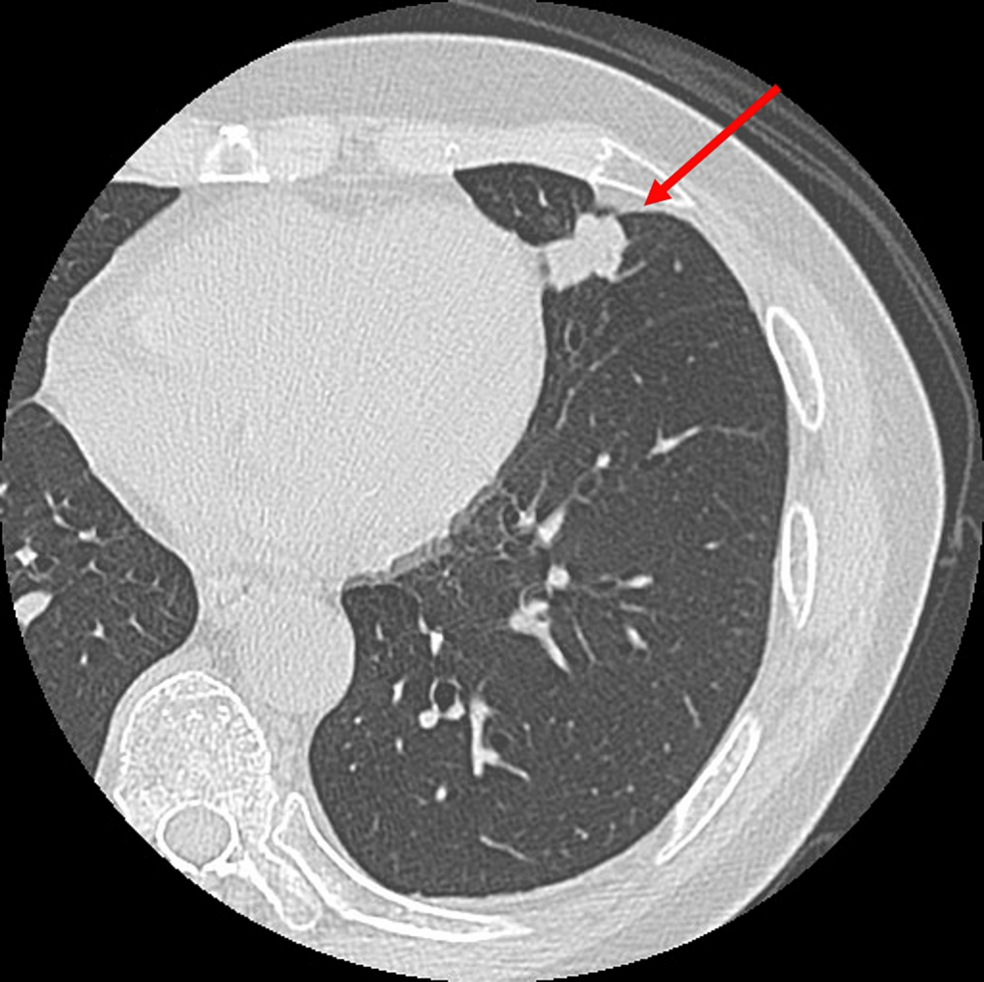
Chest CT A chest CT showed a tumor in the lingual region of the left lung with a long diameter of 20 mm (red arrow).

The patient was an 81-year-old male with no smoking history. A chest CT revealed a tumor with a long diameter of approximately 20 mm in the lingual region of the left lung (Figure 1).

**Figure 2:**
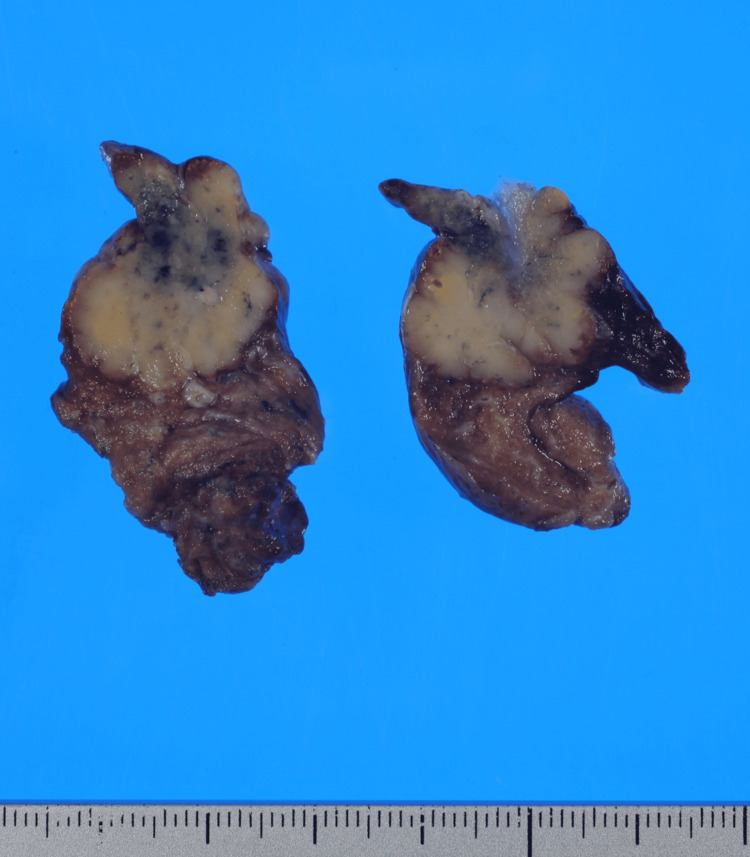
Gross findings of the resected tumor The cut surface of the tumor measuring 23 x 20 mm was well-demarcated and grayish-white in color.

A whole-body PET-CT examination showed abnormal radioactive accumulation only in the tumor site and not in others. Those findings suggested a primary lung carcinoma (cT1cN0M0, cStage IA3), so partial resection of the upper lobe of the left lung was performed. Resection of lymph nodes was not performed to reduce surgical stress because PET-CT showed no lymph node metastasis and the patient was elderly and had poor pulmonary function. At 16 months after the operation, the patient was alive with no recurrence. The tumor was 23 x 20 mm in size and well-demarcated, with the cut surface having a grayish-white appearance (Figure 2).

**Figure 3:**
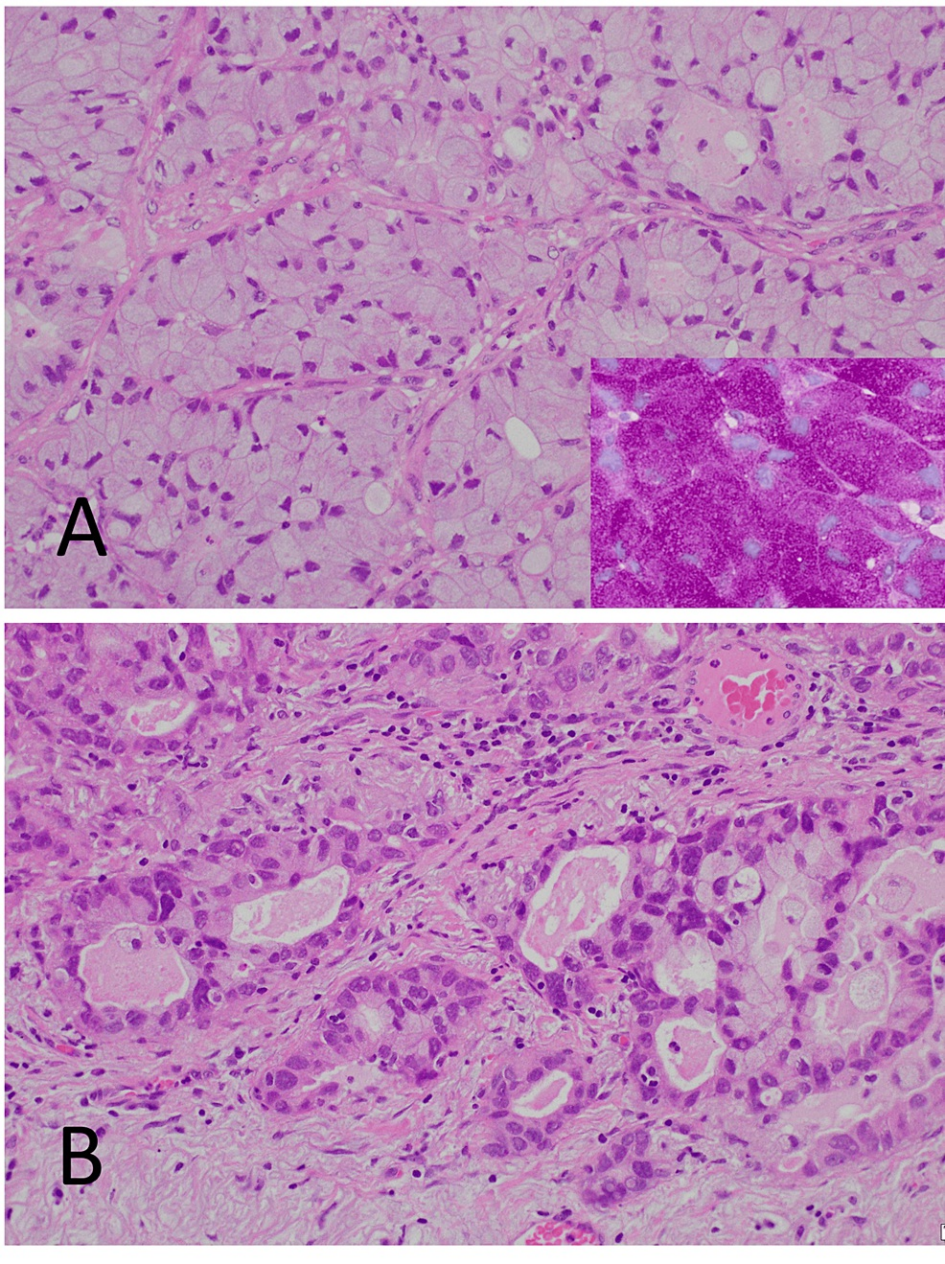
Histopathological findings (H&E staining) (A) A signet-ring cell carcinoma component is shown. Signet-ring cells have a crescent-like nucleus and abundant cytoplasm, which produces solid cell nests. Results of periodic acid Schiff (PAS) staining were strongly positive for diastase-resistant cytoplasm (inset). (B) The acinar adenocarcinoma component is shown. All microscopic photos were taken at 20x objective. H&E staining: hematoxylin and eosin staining

Histopathological findings showed that the tumor was composed of two components: 95% SRCC (Figure 3A) and 5% acinar adenocarcinoma (Figure 3B). The SRCC cells possessed a crescent nucleus displaced by abundant cytoplasmic mucin that was strongly diastase-resistant and positive in periodic acid Schiff (PAS) staining results (Figure 3A).

**Figure 4:**
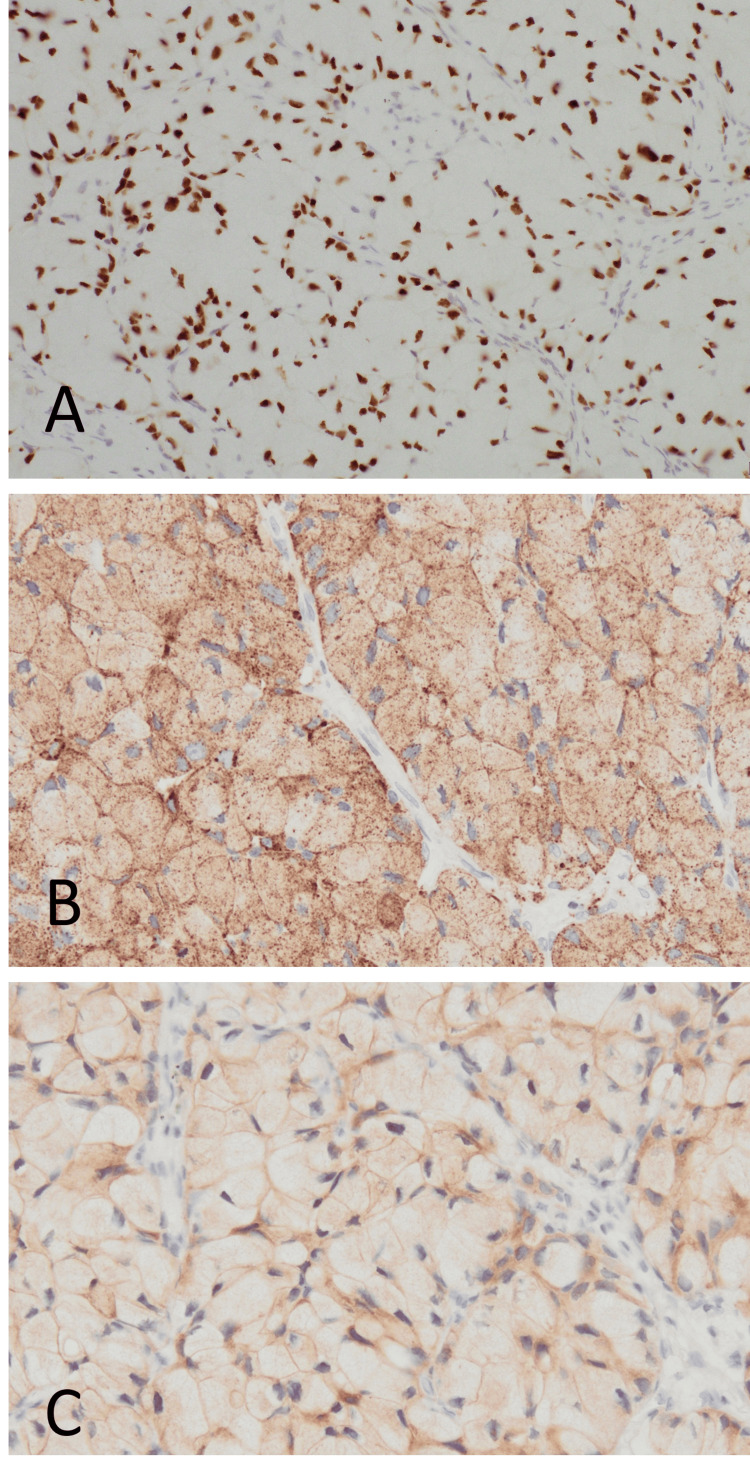
Immunohistochemical analysis Immunohistochemical staining for (A) TTF1, (B) Napsin A, and (C) *ALK*. In signet-ring cell carcinoma cells, TTF1, Napsin A, and *ALK* were positive in the nucleus, cytoplasm, and cytoplasmic membrane, respectively. All immunohistochemical microscopic photos were taken at 20x objective.

Approximately 95% of the tumor was occupied by the SRCC component, with the SRCC cells positive for thyroid transcription factor (TTF)-1 (SPT24) (Leica Biosystems, Deer Park, IL, USA) (Figure 4A) and Napsin A (IP64) (Nichirei Biosciences, Tokyo, Japan) (Figure 4B). To detect *ALK* gene rearrangement, immunohistochemical staining for *ALK* and fluorescence in situ hybridization (FISH) were performed. *ALK* staining was done with a 50-fold diluted antibody (clone ALK1; Dako, Grostrup, Denmark) using a Leica Bond III fully automated immunohistostainer (Leica Biosystems), which showed SRCC cells positive for *ALK* (Figure 4C).

**Figure 5:**
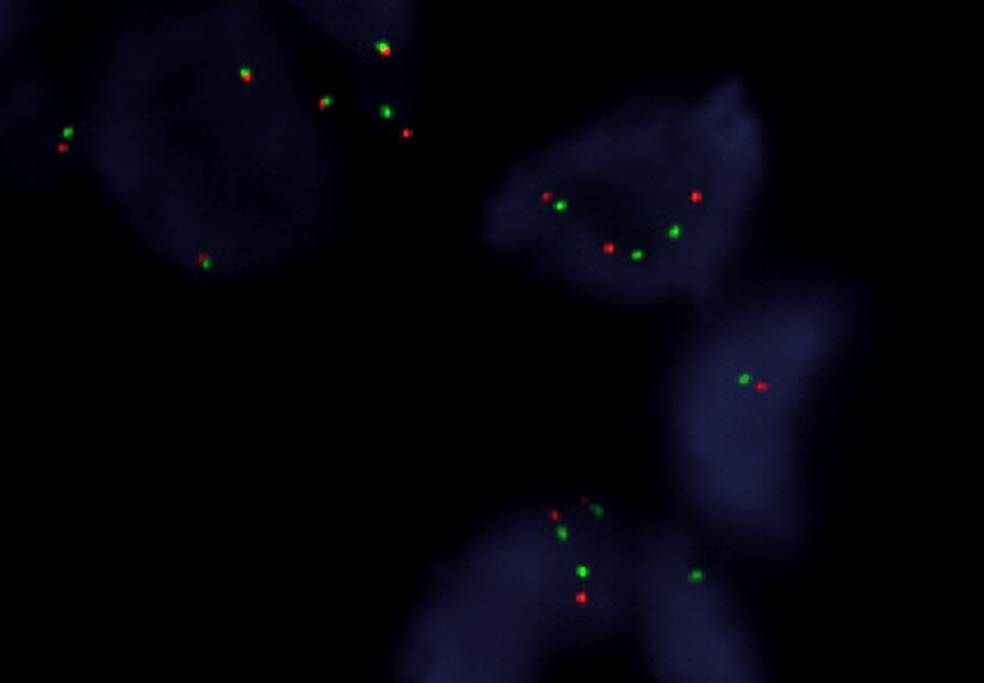
ALK FISH analysis Dual color break-apart FISH analysis showed separation of green and orange signals, indicating *ALK* gene rearrangement.

FISH was performed using a Vysis ALK Break Apart FISH probe kit (Abbott Diagnostics Medical Co., Tokyo, Japan) and analysis findings showed *ALK* gene rearrangement (Figure 5).

Use of an AmoyDx® Pan Lung Cancer PCR Panel (LC-SCRUN-Japan, Tokyo, Japan) for analysis revealed no other driver gene mutations, including the EGFR, KRAS, BRAF, MET, ROS1, NTRK, and RET genes.

## Discussion

Adenocarcinomas with an SRCC component often occur in various organs, including the stomach, colon, esophagus, and pancreas, while lung metastasis is also common [7, 8]. Therefore, it is important to distinguish a primary lung adenocarcinoma with an SRCC component from a metastasized adenocarcinoma with that component. In the present case, PET-CT showed no abnormal radioactive accumulation except in the lung tumor site, while carcinoma cells were positive for both TTF-1 and Napsin A, known markers of lung adenocarcinoma. Those findings indicated an adenocarcinoma with an SRCC component as a primary lung carcinoma in the present case.

Histology findings in the present case, excluding the SRCC component, indicated an acinar adenocarcinoma. Tsuta et al. [2] reported results of 39 cases of primary lung carcinoma with an SRCC component and noted that the histology of the tumor except for the SRCC component was acinar adenocarcinoma in 27 cases, mixed bronchioloalveolar and acinar adenocarcinoma in nine, and adenosquamous cell carcinoma in two, while in one case a pure SRCC was found, suggesting that the presence of an SRCC component is associated with an adenocarcinoma component. Nevertheless, the histogenesis of SRCC cells remains controversial [4].

Tsuta et al. [2] reported that lymph vessel invasion occurs more frequently in lung carcinomas with an SRCC component that occupies more than 50% of the tumor as compared to those without an SRCC component. Furthermore, their study found that the five-year survival rate for patients with a lung carcinoma with an SRCC component occupying more than 50% of the tumor was worse than that for patients with such a carcinoma without an SRCC component or with that component occupying less than 50% of the tumor. Although metastasis was not detected in the present case until 16 months after the operation, their report indicates the requirement of close follow-up examinations.

Immunostaining for *ALK* and FISH analysis (break-apart assay) supported the presence of *ALK* gene rearrangement in the present case, which is in agreement with previous reports of the strong association of an SRCC component in lung carcinomas with *ALK* gene rearrangement [6, 9-11]. Crizotinib, a small-molecule tyrosine kinase inhibitor, has been shown to have clinical superiority as compared to standard chemotherapy in patients treated for a tumor with an *ALK* gene rearrangement. Therefore, that is considered to be applicable for metastasis in the present patient.

## Conclusions

Presented here are details of our experience with a very rare case of primary lung adenocarcinoma, with 95% of the tumor occupied by an SRCC component. Furthermore, findings showed an *ALK* gene rearrangement in the tumor. The prognosis of the present patient may be poor because the SRCC component occupies most of the adenocarcinoma. Careful follow-up examinations of the patient will be necessary, and should recurrence be noted, the use of an *ALK* inhibitor may be beneficial.
